# What Causes Bilateral Pleural Effusion: A Case Report

**DOI:** 10.1111/crj.70055

**Published:** 2025-02-26

**Authors:** Miaojuan Zhu, Shuaiyu Lin, Yifei Chen, Jiong Yang, Hanxiang Nie

**Affiliations:** ^1^ Department of Respiratory and Critical Care Medicine Renmin Hospital of Wuhan University Wuhan China; ^2^ Department of Respiratory and Critical Care Medicine Zhongnan Hospital of Wuhan University Wuhan China

**Keywords:** case report, pericardial volume‐pressure curve, PET/CT, pleural effusion, tuberculous pericarditis

## Abstract

**Background:**

Tuberculous pericarditis begins with fibrinous and hemorrhagic pericarditis, followed by pericardial effusion, then pericardial hypertrophy, which may turn into subacute or chronic stage, and partly develop into pericarditis. Early diagnosis and treatment have very important clinical significance.

**Case Summary:**

We present a case of an 82‐year‐old man with a known history of hypertension who was admitted for pleural effusion. CT scan of the chest showed findings of pleural effusion. An echocardiographic study during admission revealed a small amount of pericardial effusion (~1.2 cm in thickness). A whole‐body positron emission tomography‐computer tomography (PET‐CT) scan was then performed and showed a slightly increased fluorodeoxyglucose uptake in the entire pericardium considering tuberculosis. He was started on antituberculosis (TB) medications and tolerated them well. Follow‐up echocardiographic study showed no re‐accumulation of pleural effusion and pericardial fluid.

**Conclusion:**

Transudative–exudative pleural effusion may be one of the clinical manifestations of tuberculous pericarditis. (1) Bilateral leaking pleural effusion may be the early clinical manifestation of tuberculous pericarditis; (2) PET/CT in the diagnosis and efficacy evaluation of tuberculous pericarditis is valuable; and (3) the central venous pressure may be an indicator of choice for treatment of tuberculous pericarditis.

## Graphical abstract

Transudative–exudative pleural effusion may be one of the clinical manifestations of tuberculous pericarditis. PET/CT in the diagnosis and efficacy evaluation of tuberculous pericarditis is valuable, and the central venous pressure may be an indicator of choice for surgical treatment of tuberculous pericarditis.

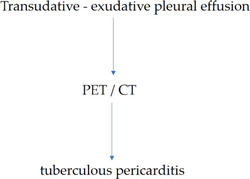

**Abbreviations**
ADA: adenosine deaminase
ALB: albumin
CT: computed tomography
CVP: central venous pressure
DNA: deoxyribonucleic acid
ECG: electrocardiogram
ESR: erythrocyte sedimentation rate
HIV: acquired immune deficiency syndrome
LDH: lactate dehydrogenase
PET‐CT: positron emission tomography‐computer tomography
PPD: tuberculin purified protein derivative
TB: tuberculosis
WBC: white blood cell

## 1 Case Report

An 82‐year‐old elderly male patient was admitted with a 10‐day history of cough, night sweats, fatigue, poor appetite fever, and chest pain, with the afternoon fever, and the a temperature fluctuation of 37.5°C–38.5°C. The patient reported no dyspnea, sensation of pressure in the chest, night sweats, fatigue, poor appetite and weight loss. The patient himself took cefixime, azithromycin, and moxifloxacin, the cough improved after taking the drug, and other symptoms did not change. He had a previous history of hypertension for more than 4 years. Initial vital signs were temperature, 37.5°C; respiratory rate, 20 breaths/min; heart rate, 70 beats/min; BP, 144 mmHg systolic and 74 mmHg diastolic; oxygen saturation, 97% on air; chest examination revealed dullness to percussion at the left and right base with reduced air entry on auscultation. Abdominal examination was normal, and there was no edema in both lower limbs. Electrocardiogram (ECG) showed a sinus rhythm of 75/min.

Blood test results were recorded in Table 1. And all tumor and autoimmune markers (ANA + ENA + ACA, ANCA spectrum and GBM antibody) were unremarkable. Tuberculin purified protein derivative (PPD) test, serum tuberculosis antibody, sputum bacterial culture, sputum acid‐fast staining, and acquired human immunodeficiency virus immune deficiency syndrome (HIV) antibody were negative.

**TABLE 1:** Blood test results.

Blood test items	Result	Range
WBC	9.21	3.5‐9.5 × 109/L
Monocytes count	1.02	0.1–0.6 × 109/L
ALB	37.4	40–55 g/L
ESR	30	0–20 mm/h
CRP	73.1	0–10 mg/L
LDH	283	125–243 U/L

Abbreviations: ALB, albumin; CRP, C reactive protein; ESR, erythrocyte sedimentation rate; LDH, lactate dehydrogenase; WBC, white blood cell.

**FIGURE 1:**
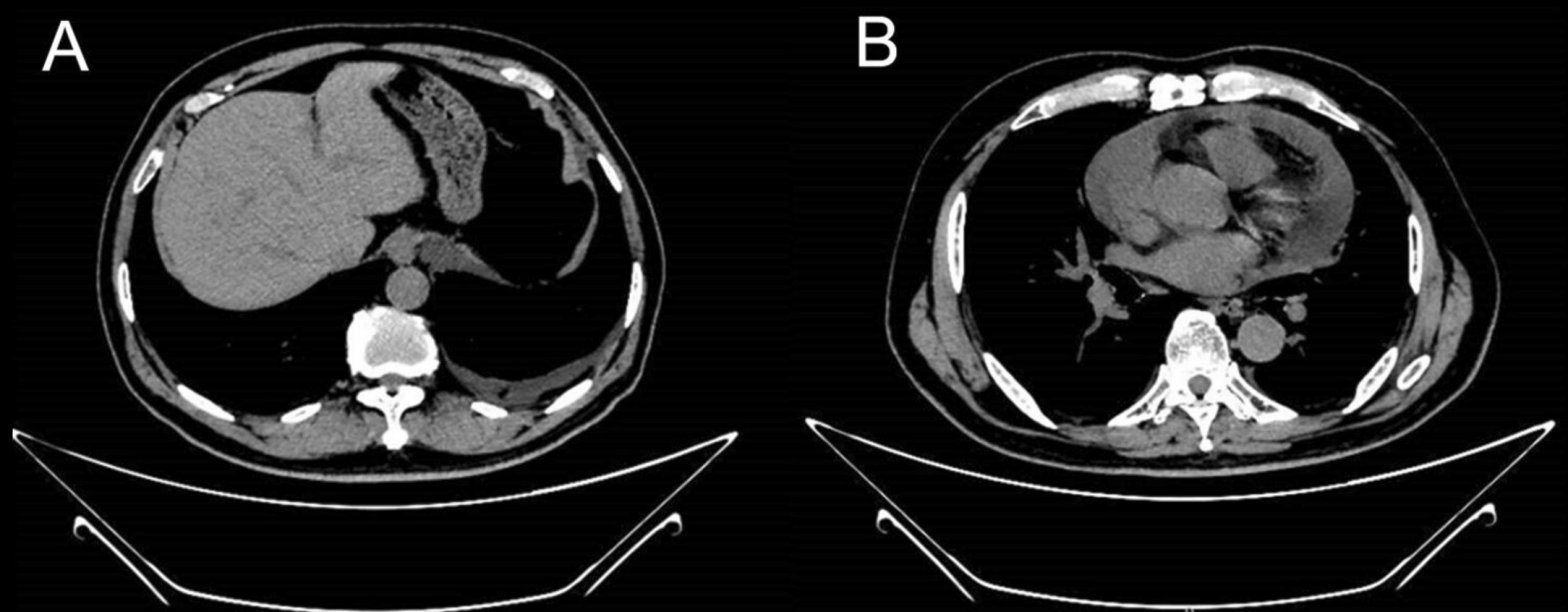
(A,B) Observation of the thorax by computed tomography (CT) showed bilateral pleural effusion and pericardial effusion.

Observation of the thorax by computed tomography (CT) showed bilateral pleural effusion and pericardial effusion (Figure 1). The results of pleural effusion analysis were shown in Table 2. And pleural fluid culture, mycobacterium tuberculosis deoxyribonucleic acid (DNA), acid‐fast staining, and tumor markers were all unremarkable. The cells in the fluid of the pleural effusion are mainly lymphocytes. We performed a metagenomic detection of pathogenic microorganisms (mNGS) in pleural effusion, and no bacterial, fungal, and viral infections were found.

**TABLE 2:** Test results for pleural effusion.

Test items	Result	Range
Total cells	342 cells/μL	—
Mononuclear cells	91.8%	—
Multinucleated cells	8.2%	—
Glucose	7.51	3.9–6.1 mmol/L
LDH	187	230–460 U/L
ADA	3	0–18 U/L
Protein	30.2 g/L	20–40 g/L
ALB	18.4 g/L	—
Cl	111.5	110–130 mmol/L
Rivalta test	Negative	—

Abbreviations: ADA, adenosine deaminase; ALB, albumin; Cl, chlorine; LDH, lactate dehydrogenase.

**FIGURE 2:**
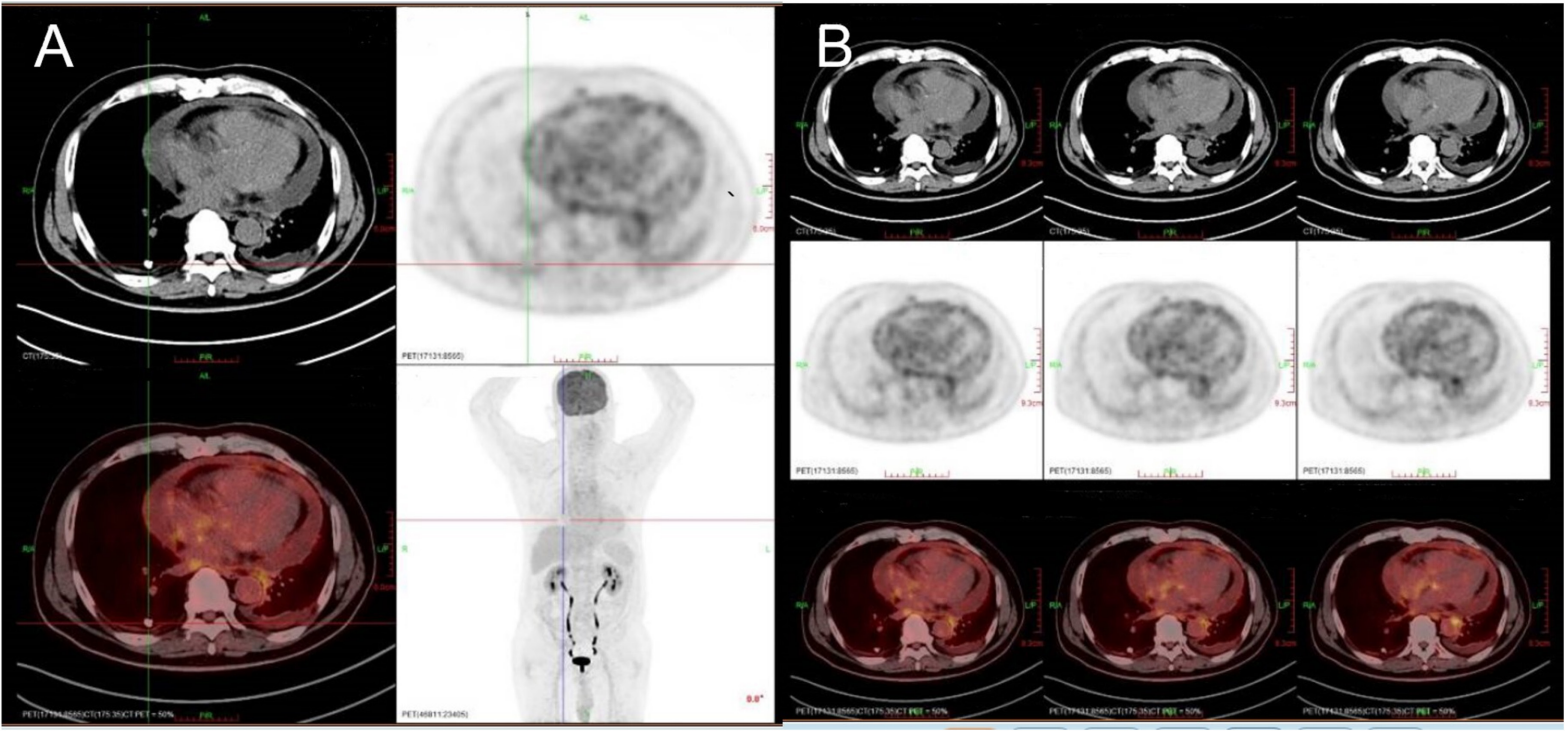
(A,B) PET/CT showed pericardial effusion with slightly increased metabolism along the pericardium.

Echocardiography revealed a medium pericardial effusion (~1.2 cm in thickness) but with no tamponade effect. There is no inferior vena cava (IVC) and/or biatrial dilation, Respirophasic interventricular septal shift or septal bounce (Table 3). PET/CT showed pericardial effusion with slightly increased metabolism along the pericardium, considered TB infection (Figure 2). To determine the nature of the pericardial fluid, pericardiocentesis was further performed. The results of pericardial fluid analysis were shown in Table 4. We also performed a metagenomic detection of pathogenic microorganisms (mNGS) in pericardial effusion, and no bacterial, fungal, and viral infections were found.

**TABLE 3:** Indicators of cardiac ultrasound.

Index	Numerical value	Reference values
Ascending aorta (AAO)	3.2 cm	2.0–3.4 cm
Left atrium (LA)	3.4 cm	2.2–3.8 cm
Left ventricle (LV)	4.5 cm	3.6–5.3 cm
Interventricular septum (LVS)	1.1 cm	0.7–1.1 cm
Left ventricular anterior wall (LVPW)	0.9 cm	0.7–1.1 cm
Right atrium (RA)	2.9 cm	2.2–2.4 cm
Pulmonary artery (PA)	2.1 cm	1.4–2.6
Fractional shortening (FS)	38%	> 25%
Ejection fraction (EF)	68%	50%–75%

**TABLE 4:** Test results for pericardial fluid.

Test items	Result	Range
Total cells	2096 cells/μL	—
Mononuclear cells	45%	—
Multinucleated cells	55%	—
Glucose	5.46	3.9–6.1 mmol/L
LDH	709	230–460 U/L
ADA	15	0–18 U/L
Protein	48.9 g/L	20–40 g/L
ALB	25.5 g/L	—
Cl	107.6	110–130 mmol/L
Rivalta test	Positive	—

Abbreviations: ADA, adenosine deaminase; ALB, albumin; Cl, chlorine; LDH, lactate dehydrogenase.

And pericardial fluid culture, mycobacterium tuberculosis DNA, acid‐fast staining, and tumor markers were also negative. The cells in the pericardial fluid are mainly neutrophils and lymphocytes. The patient had central venous pressure (CVP) of 14 cm H_2_O before pericardiocentesis. We used the manual measurement method to measure the CVP. The main operation process is to connect the catheter, the infusion device, and the manometer with a three‐way tube. The patient is in a supine position, and the 0 point of the manometer is at the same level as the right atrium. The normal saline in the infusion device enters the manometer, closes the clamp of the infusion device, opens the clamp on the catheter, and observes that the liquid in the manometer drops to a certain level and no longer drops, that is, the CVP measured manually.

Further investigation was performed to search for the underlying cause of pleura and pericardial effusion.

**FIGURE 3:**
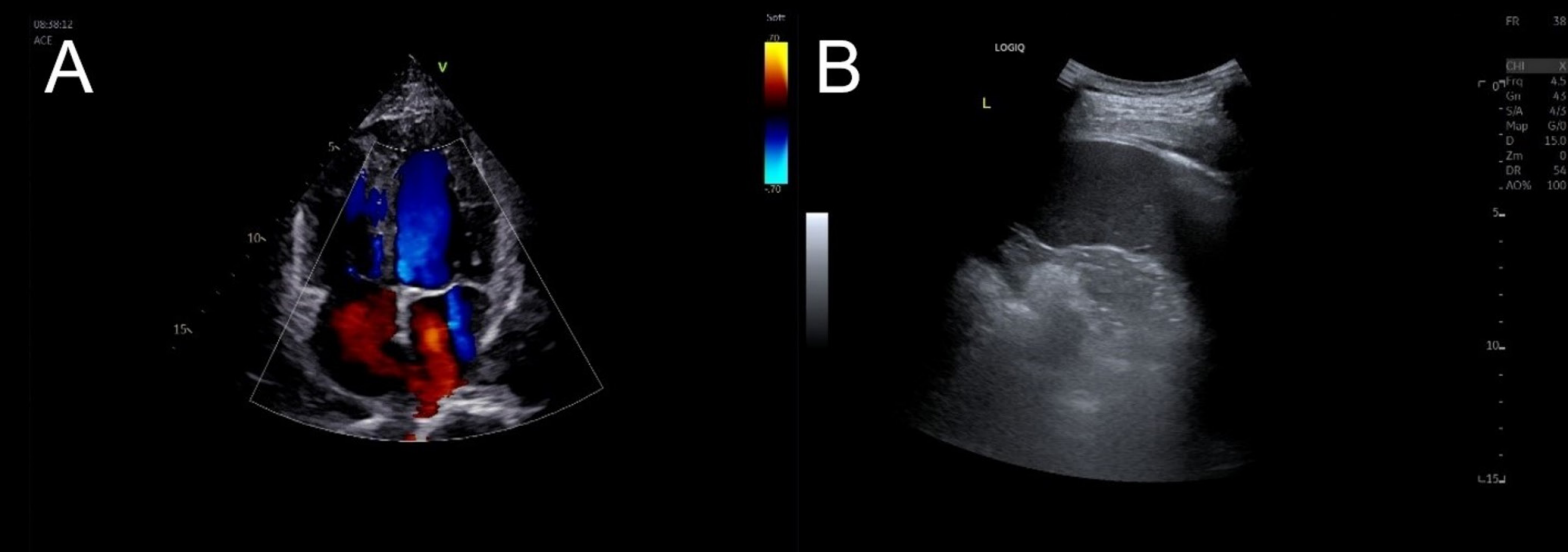
(A) Cardiac ultrasound did not show pericardial effusion; (B) thoracic ultrasound had no signs of pleural effusion.

We conducted a multidisciplinary discussion and diagnosed it as tuberculous pericarditis after a multidisciplinary team discussion. He received anti‐infection (moxifloxacin 400 mg QD) and antituberculosis chemotherapy (isoniazid 0.3 g QD, rifampicin 450 mg QD, and ethambutol 0.75 g QD). He was discharged after symptoms such as chest pain disappeared and continued to receive antituberculosis treatment and observation in the outpatient department of our hospital. At present, the patient has received antituberculosis chemotherapy for 8 months and is in good condition. The review suggests that pleural effusion and pericardial effusion are completely absorbed (Figure 3).

## 2 Discussion

Tuberculous pericarditis is caused by *Mycobacterium tuberculosis* . And it is found in 1% to 2% of people who have pulmonary tuberculosis in endemic areas. Approximately 80 000–160 000 new cases of tuberculous pericarditis occur each year in Africa and East Asia [1]. Diagnosing tuberculous pericarditis is not easy in many cases. Patients with tuberculous pericarditis will have serious complications without early diagnosis and treatment, and the mortality rate of untreated cases is very high [2].Tuberculosis spreads to the pericardium via three main mechanisms. It can spread retrogradely from mediastinal, peritracheal, and peribronchial lymph nodes, via the hematogenous route during primary tuberculosis and by direct spread from lung parenchyma or pleural involvement [3].

Pleural effusion occurred in 40% to 50% of people with tuberculous pericarditis. The hypothesis mechanism of transudative pleural effusion in constrictive pericarditis patients is that ventricular diastolic dysfunction is caused by pericardial thickening, with increased pressure in the atria. Increased left atrial pressure leads to the associated pulmonary vein hypertension. Elevated pulmonary venous and capillary pressure impedes thoracic venous drainage, leading to pleural effusion. On the other hand, increased right atrial pressure blocks fluid return by interfering with pleural and pulmonary lymphatic drainage. Right ventricular dysfunction will lead to systemic venous congestion and ascites. Ascites can enter the thoracic cavity through the diaphragmatic fissure, causing exudative pleural effusion [4].

However, increased atrial pressure leading to pleural effusion is not appropriate in all cases. Tomaselli et al. [5] found increased atrial pressure in 20 patients with constrictive pericarditis after cardiac catheterization. In these patients, increased atrial pressure levels were not associated with the presence of pleural effusion. That is to say, the mean values of right atrial and pulmonary capillary wedge pressure in patients with and without pleural effusion were similar. Tomaselli et al. also found that 75% of patients underwent thoracentesis with exudative pleural effusions. When constrictive pericarditis leads to right ventricular dysfunction, the average CVP is 20.42 ± 0.50 cm H_2_O [6].

The case echocardiography we report did not find ventricular diastolic dysfunction, and the measured CVP was 14 cm H_2_O. There was no imaging evidence of pericardial thickening and calcification. Through comprehensive analysis of relevant evidence, our final diagnosis is tuberculous pericarditis, not tuberculosis constrictive pericarditis. Jorquera‐Román et al. reported a case of transudate pleural effusion in a patient with tuberculous pericarditis. But this patient's chest CT revealed a massive pericardial effusion (~3.5 cm in thickness). We report the occurrence of transudative pleural effusion in patients with tuberculous pericarditis without ventricular diastolic restriction. Moreover, pleural effusion is the only extracardiac manifestation of patients other than moderate pericardial effusion.

In this report, we propose a hypothesis about the mechanism of transudative–exudative pleural effusion in patients with tuberculous pericarditis. The patient we report had a small amount of pericardial effusion, consistent with the initial stage of chronic exudation of the pericardial pressure–volume curve [7]. The patient we report had a small amount of pericardial effusion, consistent with the initial stage of chronic exudation of the pericardial pressure–volume curve. Low increase in pericardial pressure on left ventricular diastolic impact, so that pulmonary vein and capillary pressure increased slightly, forming a small amount of leakage of pleural effusion. Of course, the patient's right ventricular diastolic was also affected, but the question was why the patient had pleural effusion, but there was no systemic venous congestion, such as ascites. Anatomically, the pulmonary vein returns to the left atrium, and the systemic venous blood returns to the right atrium. From the perspective of respiratory physiology, when inhaling, the negative pressure of the pleural cavity increases, which helps the systemic vein return to the right atrium, which can partially offset the effect of the small increase in pericardial pressure on right heart relaxation. However, for pulmonary veins, when inhaling, the negative pressure of the chest makes the lung tissue and pulmonary vessels dilate. To better gas exchange, the amount of blood entering the pulmonary vessels increases, and the pressure of the pulmonary veins increases. At the same time, the left displacement of the interventricular septum to the left ventricle leads to a decrease in left ventricular volume, which further amplifies the effect of pericardial pressure. Wiener‐Kronish et al. reveled that in patients with congestive heart failure, an elevated left atrial pressure is closely correlated with the presence of pleural effusions, whereas concurrent elevation of right atrial pressure is not associated with the presence of pleural effusions. These hypothesis mechanisms explain that in patients with pleural effusion but no systemic venous congestion. As the volume of pericardial fluid increases, the pericardial pressure curve rises to a steeper stage, and the negative pressure of the pleural cavity cannot play a key role in offsetting right ventricular diastolic restriction. Further patients will have ascites and lower extremity edema. However, the range of compensatory pressure generated by respiration is currently uncertain and needs further study. Pleural effusion may be the earliest symptom in patients with tuberculous pericarditis with a small amount of pericardial effusion. If our hypothesis is established, it can provide a new direction for the clinical diagnosis of tuberculous pericarditis. There is another possibility is that the production of pleural effusion has nothing to do with the heart. Poor nutritional status in elderly patients with reduced serum albumin, further leading to transudative pleural effusion. According to animal studies, pleural effusion itself can cause an increase in CVP [8]. The further findings need to be explored.

Another major objective of our case report is to discuss the early diagnostic value of PET/CT in patients with tuberculous pericarditis. The PET/CT of the patient showed pericardial effusion with slightly increased metabolism along the pericardium. It is not safe to give antituberculosis chemotherapy based on imaging evidence alone. Then, we asked the cardiac surgeon for a pericardial puncture. Pericardial effusion was consistent with tuberculosis changes and was finally diagnosed as tuberculous pericarditis.

In the current study, PET‐CT provides valuable information about the degree of pericardial inflammatory activity without the need for invasive diagnostic procedures to confirm pericardial involvement and disease activity. Lee et al. [9] reported a case of tuberculous pericarditis with the whole pericardium 18F‐FDG uptake. In addition, the usefulness of FDG‐PET in monitoring the response of tuberculous pericarditis to treatment has been demonstrated in current cases. Testempassi et al. [10] found that two patients with tuberculous constrictive pericarditis had 18F‐FDG uptake in the pericardium on PET/CT (Case 1 [max SUV 13.9] vs. Case 2 [max SUV 16.5]), and after antituberculosis treatment, the 18F‐FDG uptake rate decreased, indicating that PET/CT can be used not only as a diagnostic tool but also as a means of efficacy evaluation. Ozmen et al. also demonstrated that PET/CT can be used to assess the efficacy of antituberculosis chemotherapy, which can reflect changes in the degree of inflammation [11]. A retrospective study found that the mean pericardial thickness (SD) and max SUV of acute tuberculous pericarditis were significantly higher than those of acute idiopathic pericarditis (5.1 [1.0] vs. 3.4 [0.9], *p* < 0.05; 13.5 [3.9] vs. 3.0 [0.7], *p* < 0.05) [12]. In conclusion, PET/CT may be an important non‐invasive method for the diagnosis of active diseases and help to deal with extrapulmonary mycobacterial infections including pericarditis. In addition, our report provides relevant evidence that 18F‐FDG‐PET is meaningful for the diagnosis of tuberculous pericardial disease.

The CVP of the patient we reported was 14 cm H_2_O. In the previous discussion, we suggested that patients with constrictive pericarditis had an average CVP of 20.42 ± 0.50 cm H_2_O when the ventricular function was limited and had to undergo pericardial surgery. Whether the value of CVP may be an important indicator of the choice of surgical treatment for tuberculous pericarditis requires a large number of retrospective and prospective studies to further confirm. If a large amount of research data and evidence‐based analysis can be obtained, perhaps many patients with tuberculous pericarditis can avoid surgical treatment.

## 3 Conclusions

When patients with pericarditis have unexplained transudative–exudative pleural effusion as the main clinical manifestation, we cannot rule out tuberculosis infection, especially tuberculous pericarditis. PET/CT in the diagnosis and efficacy evaluation of tuberculous pericarditis is valuable. The CVP may be an indicator of choice for surgical treatment of tuberculous pericarditis.

## Author Contributions

Miaojuan Zhu is the first author of the article. She is responsible for writing the whole article and grasping the purpose of the full text. Shuaiyu Lin is responsible for all relevant tables and pictures in the article. Yifei Chen is responsible for the search and entry of references. Hanxiang Nie and Jiong Yang, as the corresponding authors, are responsible for the proofreading and revision of the full text. As the person responsible for communication, Hanxiang Nie is responsible for the authenticity of this article.

## Ethics Statement

This study was approved by the Ethics Committee of Zhongnan Hospital of Wuhan University (2022255K). Consent was obtained by the patient in this study.

## Conflicts of Interest

The authors declare no conflicts of interest.

## Data Availability

Data sharing not applicable to this article as no datasets were generated or analysed during the current study.
